# Extra Supplement—The Nursing Mirror

**Published:** 1890-09-06

**Authors:** 


Being the Extra Nursing Supplement of "The Hospital" Newspaper.

All Contributions for this Supplement should be addressed to the Editor, The Hospital, 140, Strand, London, W.C., and should have the word "Nursing" plainly written in left-hand top corner of the envelope.

### En Passant.

The Work of the Private Nurse.—"The nurse should be willing to do everything in her power to assist her patient to recover health. If the mother of the house is ill, and there is no other woman to care for the children, the nurse should be willing to wash their faces and darn their stockings; but in a family where there are many servants it is not the nurse's duty to do this work. In each separate case the nurse should have tact and judgment enough to find her place and her duty. Still she must show some consideration for her own body and soul. Many nurses, prematurely worn out by injudicious enthusiasm during their earlier career, are now obliged to sift their cases. When such refuse to answer a call let others 'judge not.'"—*The Nightingale.*

Louisa Alcott as Hospital Nurse.—"A strange life—all night hovering in a red rigolette over the slumbering sons of man. I liked it, and found many things to amuse, instruct and interest me. I would have given much to have possessed the art of sketching, for many of the faces became wonderfully interesting when unconscious. Some grew stern and grim, the men evidently dreaming of war as they gave orders, groaned over their wounds, or cursed the rebels vigorously; some grew sad and infinitely pathetic, as if the pain, borne silently all day, revenged itself by now betraying what the man's pride had concealed so well. Often the roughest grew young and pleasant where sleep smoothed the hard lines away, letting the real nature assert itself; many almost seemed to speak and I learned to know these men better by night than through any intercourse by day. Sometimes they disappointed me, for faces that looked merry and good in the light seemed bad and sly when the shadows came, and though they made no confidence in words I read their lives. A few talked busily."

Catholic Nurses.—A study of the nursing question in all lands, through all ages, impresses the student very vividly with the wondrous nursing work done in early times by the nuns. They were the pioneers, they bore the heat and burden of the day, but now they are being superseded. How is this? Undoubtedly it is because the sisters of charity are so seldom "trained" in the knowledge and skill which the doctor now demands of his hand-maid in the sick room. A certain amount of practical knowledge every Roman Catholic sister of an active order receives, but why should there not be lectures on anatomy, physiology, and general nursing in the novitiate of every nursing community? The nuns who keep schools in Italy have been obliged to learn drilling and gymnastics, that they may teach them to their pupils: it would be a no more worldly pursuit for the nuns to read Quain and Huxley; and surely a skeleton would be an appropriate ornament for their refectory. Lay nurses did not scorn to learn all they could from the *Sœurs de Charité.* Miss Nightingale worked under the celebrated order of St. Vincent de Paul. Religious nurses then might well at this juncture study the Nightingale system of training at St. Thomas's, or the system of district nursing of the Metropolitan and National Association. Without being Roman Catholics we can yet regret that the life-long devotion of many of the sisters is not doubled in practical value by increased training in the skilled work her hands find to do.

The Melbourne Commission.—In the multitude of councillors there is wisdom, so here is further evidence from Australia. Dr. Rowan said: "Female nurses were 90 per cent. better than men, as you could always rely upon having instructions carried out, while men were more prone to neglect their duties. Eight hours a day was sufficiently long for a hospital nurse to be on duty. No matter how robust a woman was, if she remained in an atmosphere of sickness for long periods at a time, she was bound to get sick herself. He had always thought it a hardship that women who acted as nurses should be expected to work for longer periods than men. Eight hours a day was fixed as the standard period of employment in the colony. Why then should women not participate in the system? He knew of nurses competent to use the hypodermic syringe, but he had never known of their doing so without instructions. Six months' training had to be undergone by nurses at the Women's Hospital before they obtained certificates. Trained nurses certainly obeyed their instructions better than untrained ones."

The Nurse-Dolls.—The dolls are now unpacked and temporarily arranged in a case at The Hospital office, 140, Strand. Any nurse calling between the hours of ten a.m. and four p.m. can see them. The case which has been made for the dolls is rather small for showing them to advantage, so that when our increasing work makes it necessary to seek larger offices once more, we hope to get a second case made which will allow room for each doll to show off her special beauties. In the meanwhile, however, those nurses, or would-be nurses, who so constantly write to us for cap patterns and other information connected with nursing uniforms, can see every style of cap and apron on the dolls, and can copy those they like best.

Our Duties to the Dying.—A discussion has been raised in America as to how far a nurse may go in administering spiritual comfort to the dying patient under her charge. It is always a sore subject this; whether it is wise or no to "prepare" the patient to die. Oliver Wendell Holmes says: "Nothing is clearer than that the merciful Creator intends to blind most people as they pass down the dark valley. Without very good reasons, temporal or spiritual, we should not interfere with His kind arrangements. It is the height of cruelty and the extreme of impertinence to tell your patient he must die, except you are sure that he wishes to know it, or that there is some particular cause for his knowing it." But then there are some people who believe in a death-bed repentance, and think no nurse or doctor ought to let the last unconsciousness fall on a patient without warning. The case of the thief on the cross is generally given as proof of the power of a late repentance. But the prayer of the thief was spontaneous—it was no preaching, no warning, which wrung that cry from him: was it not rather the sight of suffering borne in silence? It is not by exhortation or rebuke so much as by example that you can speak to the soul which is already withdrawing from the earth. The nurse's duty is not to preach, it is to minister. She has responsibility enough on her shoulders without being troubled with the argument that it is her duty to warn the dying. Let others—the doctor, the clergyman, or the family—settle that question, and let the nurse remember that there is an unspoken language by which the best and most powerful preaching in this world is done.

### Sketches of Insanity.

By Francis H. Walmsley, M.D. (Senior Assistant Medical Officer of Leavesden Asylum).

### IX.—WEAK MIND.

In *Idiots* and *Imbeciles* the state of imperfection of the nervous system renders them slow in perceiving impressions from without; their senses are dulled, their sensibility obtuse, and thus they are capable of but a slight degree of attention; they see badly, hear badly, feel badly; this defect of observation and attention is the cause of the inaptitude of idiots for education, and extends so far in some instances as to prevent the acquisition and conservation of the ordinary acts that go to make up the routine of life.

*Idiocy* in its lowest form combines the extreme of bodily deformity with an existence purely vegetative, the mind being almost wholly absent; such idiots seem devoid even of sensation, and would perish if not attended to. The actions of these beings appear to depend on mere animal instinct, they recognize no one, remember no one; the unfortunate subject sees nothing, feels nothing, does nothing, knows nothing; he may be blind, deaf, or dumb; or if he sees, the gaze is wandering—he sees, but he does not regard, attention is wholly absent. In a somewhat higher form there are sensations of heat and cold, of hunger and thirst, and just intelligence enough to indicate the commonest wants by signs.

A still higher class consists of those idiots who have sensation and consciousness. They are able to recognize familiar persons and objects, can move from place to place, make known their wants by gestures and sounds, or even by words imperfectly articulated. Others only utter monosyllables and certain cries, or grunt from time to time. Others again may learn to hum or sing. We may see in them a certain display of affection, a sort of gratitude towards those who nurse them and are continually with them; such subjects are susceptible of some improvement in their bodily and mental condition.

Most idiots are of very inferior physique; they are dwarfish, and exhibit a general want f symmetry, have small misshapen heads, features ill-formed and distorted, squinting eyes, large gaping mouths with thick lips, irregular teeth, sallow and unhealthy complexion, the limbs and trunk imperfectly developed, and the gait awkward and unsteady. When, in growing, idiots gain strength they exert it uselessly; thus one spends his life in pulling out and pushing in the drawer of a table; many give themselves up to a sort of balancing; others rock the body to and fro ceaselessly, or perform certain monotonous, meaningless movements.

*Imbeciles* possess the faculty of speech as distinguished from the parrot-like utterance of the few words which the idiot can learn. They are capable of some, though in most cases slight, mental culture. They have a limited power of acquiring or retaining knowledge and of recording the experiences of life. They cannot understand or appreciate the customs or laws of society, and are incapable of controlling their emotions and passions; they can invent nothing, but their power of imitation is often considerable; in this respect it is curious to see certain subjects learn a trade and gain a living.

Some display much shrewdness, and are constantly indulging in jokes. They pass for half-witted folk, whose droll behaviour and ready repartees create amusement. From this class the Court fools of antiquity and of the middle ages were derived.

Many idiots and imbeciles are capricious in temper. They display lying and thieving propensities, are often spiteful, get into mischief, are liable to violent outbursts of passion, and sometimes commit acts of atrocious cruelty; in many the senses are very unequally developed. Certain idiots insensible to all other impressions have an extraordinary taste for music, and are able to retain an air which they have once heard. In rare instances there is a memory of forms and colours, and an aptitude for drawing and painting; some show remarkable ingenuity and skill in making models of ships and houses; some can perform mental calculations of considerable difficulty. There are some imbeciles whose memories within certain limits have been very remarkable. Thus, one who showed not the slightest intelligence on any other subject, could recite correctly the name, age, date of burial, and the epitaph inscribed on the tomb-stone of all who had died during the many years he had lived in the village.

*Cretinism.*—In some of the low-lying districts of Switzerland, and in the valleys lying among the hills in other parts of Europe, there are to be met with unfortunate creatures known as *Cretins*, who combine the extreme of bodily deformity and degeneracy, with deficiency of intellect. The morbid feature by which they are chiefly distinguished is the enlargement of the throat—or goitre—and the brown or yellow colour of the skin; the body is dwarfed and squat, they are mostly under four feet, and may be little more than two and a half; they exhibit a total want or marked imperfection of the power of speech, deficient muscular energy, and inability to stand beyond a certain time. At the age of fourteen they look like little old men and women.

*Weak Mind.*—There are many individuals living at large who are affected in a certain degree; they are reputed persons of a singular wayward and eccentric character; they often display great ingenuity in giving reasons for the eccentricities of their conduct; they manifest feebleness of purpose and a facility of disposition which lays them open to be imposed upon by the artful and designing; they are always forming acquaintances with unworthy persons who find it worth their while to know and to flatter them; throughout life they are weak, wavering, and fickle; it is impossible to put any dependence in their promises; they exhibit want of self-control, inaptitude for business, disregard of duty, and lack of common honesty. Such persons are known in society as easy, good-natured, well-meaning sort of people, and if possessed of brilliant talents, as having every sense but common sense; they are too easy to be just, too thoughtless to be honest!

*Moral Insanity.*—It is difficult to say where moral depravity ends and moral insanity begins. We meet with many cases in both sexes, but especially in women whose tempers are so bad and so uncontrolled, that they commit all kinds of evil actions, treat those nearest to them in the most wicked manner, make false and malevolent accusations against them, hurting their feelings and doing them injury in the most deliberate way; they are jealous and spiteful, and delight in annoying their friends. It would seem sometimes as if a universal badness had taken possession of them, yet a badness so inexplicable that it can only be looked upon as madness.

Such persons have no scruples about falsehood, and will deny or justify everything with which they are taxed. There appears to be an entire absence of the moral sense and corresponding want of self-control. It is useless appealing to higher sentiments of which they are destitute. With great plausibility they justify the state of moral feeling under which they appear to exist. In these cases anxiety and difficulty are experienced. They are troublesome and mischievous to their friends and to the public. They are the "thorn in the flesh"—the "moral pest" of the community in which they move.

No one has lived long in the world without meeting examples of such ill-regulated beings—victims to the tyranny of their vicious organization. There is something radically wrong! What is it? Madness or badness?

(*To be continued.*)

### Everybody's Opinion.

[*Correspondence on all subjects is invited, but we cannot in any way be responsible for the opinions expressed by our correspondents. No communications can be entertained if the name and address of the correspondent is not given, or unless one side of the paper only be written on.*]

### LADY NURSES AND DOMESTIC SERVANTS.

"A SERVANT" writes: I was rather startled to read in last week's HOSPITAL that the majority of nurses are ladies. In my ignorance I had imagined otherwise. There are, I believe not a few. But "Lady (?) Nurse" is too sweeping in her bold assertion that "no one who belongs to the servants' class of to-day could pass the examinations and fill the responsible posts our nurses do nowadays." The gist of two sermons lately published flashed into my mind when I recovered the shock—"Medical Calf" and "Baby Brains." The thought was, perhaps, uncharitable, but one privilege of our nation is self-vindication. That refinement and education are indispensable in a nurse I most tenaciously hold; but why speak so disparagingly of the intellect of the "servant class of to-day?" When was education higher in the lowest strata of society than to-day? I readily admit there are women who disgrace the class, coarse and ignorant, but are even the roughest, utterly devoid of sense and feeling? The line must be drawn somewhere; all are not hopeless. "The gentler born the maiden . . . the more bound to be sweet and serviceable." It is well to mix some of the spirit of Mrs. Fry and her followers with Miss Nightingale and her followers. Can education and refinement alone endow a woman with that "power of touch" to "smooth the pillow," which small thing is rest and ease to the sick at times? or teach her to be patient, decided, and cheerful to the desperate would-be suicide, or the confirmed hypochondriac? or can education, refinement, and wealth combined, furnish that strength and presence of mind which surgeons naturally expect to be displayed at operations? There are even servants who possess these essentials. I could introduce you to ladies of education, refinement, and wealth, in whose hands "Lady Nurse" (sick) would be sorry to find herself. She accuses Miss Twining of "somewhat contemptuous" speaking. I have never read a disparaging remark on servants by Miss Twining, or anyone else in THE HOSPITAL till now. Nurses are not paupers, especially "lady nurses," and there are those among the "rare" ones whose charitable, self-denying spirit will be sore wounded if £15,000 are expended for a "Home of Rest for Nurses." When we hear of wards of hospitals being closed for lack of funds, the idea of spending that sum in another way than to open them again seems preposterous, and surely one could count on the fingers the nurses who agree with such a proposal. I believe many ladies would be happy to "house and cheer" a homeless nurse if it were only suggested to them (even though she had once ranked with servants), and who would scorn to make her feel her inferiority. There are those from the servants' class who have passed examinations successfully, and are holding responsible posts. I could introduce "Lady Nurse" to more than one; and servants will continue to enter the ranks that do exploits against sin, suffering, and death. I know one whose whole soul is set on the work, but whose duties outside hospital walls prevent her immediate entrance, and she not flying to hospital to be cured of love-sickness, but from pure love to the work and the workers. There are, and still will be, ladies, in the true sense of the word, who welcome those of inferior education and birth, with minds and hearts, and who are far too great and noble to allow of a petty thought of birth when engaged in a conflict such as hospital nurses are engaged in. Real "Lady Nurses" are far too womanly and generous to flaunt their birth and wealth before the eyes of the servant class. My parents were poor, but highly respectable; too poor to give us an education as liberal as desirable. We are not accomplished. Nevertheless we can and do mix with a stratum of society above our own, and are not spurned for so doing. Our eldest sister passed a stiff examination in one of the first London hospitals, and now holds certificate and testimonials from the authorities, as well as from high-class families; and if the writer realizes the height of her ambition, she will do better still. Our eldest brother is one of the first engineers on the London, Brighton, and South Coast Railway, and is held in high esteem by the governors of the same. Others of our family are likely to be as prosperous, though born of poor parents and started in the "servants' class of to-day." Caste is observed, in a way, as much in England as in India, and the more strictly it is observed the more it will impede the progress of a glorious work. I love my mistress, and my mistress loves me. I have served and nursed her for six years, and hope to do so another six, though she did attain to her ninetieth birthday last month. Considering the Lydia Languishing young ladies of to-day, I am rather proud to belong to the despised class of domestic servants.

### A NURSE'S HOURS.

"A. E. S." writes: It is very easy to say and write nurses have too long hours, but very difficult to give them shorter time on duty, particularly in small provincial hospitals, where there is only a charge nurse and one probationer, and where all the cleaning (except the ward floors), fire making, washing up, &c., must be done by either one or the other, and where the staff is never so punctual as in a London hospital. I am sure from 6.30 a.m. to 9 p.m. is too long for women to be in sick wards, and so long as a nurse is in the ward something will always be wanted. Any suggestions would be gladly received as to how this can be altered. We know domestic servants have, as a rule, only one evening a-week off, but their work is so varied. Nurses do grow very weary, though they most of them love their work, more or less; but now, when strong, hearty men are all crying out for eight hours' work and eight hours' play, surely it is not much for us to ask for two hours off some time during the day, leaving us still over twelve hours' work. Those who always live in a crowd alone know the pleasure of "a bit of time for onesself." Money comes in slowly, and expenses always crop up fast, and the general public spends a good deal of "gush" and romantic talk about nursing and nurses; but let them come and stand by a helpless case for an hour three or four times a-day, when it takes two nurses that time to make it clean and comfortable, and we think the "romance" would turn to "money."

### The Nurses' Bookshelf.

### A NOVEL FOR NURSES.*

WE can most heartily recommend this intetesting book to the notice of nurses, and to all who want a really good story for a holiday or leisure-time, and we may mention that the whole is contained in a single volume of moderate price—no small advantage this, we venture to think.

The interest starts from the first, and is so well sustained that it can be truthfully said that throughout the whole book not one dull page can be found. Therefore, though the treatment of the heroine by her guardian, Mr. Girdlestone, senior, is at times most harrowing to one's feelings, and makes one marvel that any human being could endure it and retain her reason, still, the story ends in the truly orthodox way with the reward of the righteous, the marriage of the heroine with her faithful lover, and the death of the elder Girdlestone in one of his own unseaworthy ships—so that in the words of the old story-books of one's youth, all who de-

* The Firm of Girdlestone. A romance of the unromantic. By A. Conon Doyle. (Chatto and Windus.)

served to do so "lived happily ever afterwards, and kept pigs."

We will not spoil the pleasure which we hope may be in store for many nurses and others, by relating, even briefly, any part of this story, but hope they will turn to the pages themselves and learn it at first hand. We may say, however, that a story which contains such characters as the two Girdlestones, father and son, both of them thoroughly unprincipled cunning men who devote all their ingenuity, to propping up their tottering African business, in doing which they do not scruple to call to their aid such trifles as over-insurance of ships, altering the "Plimsoll-mark" to enable them to over-load their unseaworthy vessels, which ship water to a dangerous extent, the formation of a "diamond corner," the attempt to obtain the fortune of their ward and swamp it in their business, a task which requires the most delicate wiles of two such rogues as the Girdlestones—all these facts skilfully woven together produce a very interesting story.

Of other characters that of Tom Dimsdale is well drawn. We are introduced to him as a young medical student at Edinburgh who is "ploughed" in his "First Professional" through not correctly diagnosing a bubble in a pond and throws over medicine for which he has little natural taste in favour of commerce, and is thus introduced as junior partner to the Girdlestone firm and becomes attached likewise to the ward, Kate Harston. The glimpse given of life in Edinburgh is a very pleasant one, and the football match between rival English and Scotch teams could only have been described by a devotee of the noble game, and will, we expect, stir the soul of many a player. Undoubtedly, however, the most interesting part in this fascinating story is where the scene is laid at the mines in South Africa, whither the junior partner goes to carry out the details of his "diamond corner."

Nor must the clever, kind-hearted Irishman, Major Tobias Clutturbuck be omitted, who shares very humble lodgings with his friend, the German clerk, until good luck and a properous widow turn up for him at one and the same time, and he is enabled to pass the rest of his life in well-earned comfort.

### Appointments.

[It is requested that successful candidates will send a copy of their applications and testimonials, with date of election, to THE EDITOR, The Lodge, Porchester Square, W.]

BIRMINGHAM AND MIDLAND SKIN AND LOCK HOSPITAL.—Miss M. A. Carter, of the Cottage Hospital, Lyme Regis, has been elected to succeed Miss Horner as matron of the Birmingham and Midland Skin and Lock Hospital. There were 32 candidates.

BROWNLOW HILL INFIRMARY.—Miss Harriet Hetherington, trained at the London Hospital, has been appointed night superintendent of this infirmary.

BOLTON DISTRICT HOME.—Miss Thomas, trained at the Temperance Hospital, and the Metropolitan and National Association, has been appointed lady superintendent of this home.

### Notes and Queries.

TO CORRESPONDENTS.—1. Questions may be written on post-cards. 2. Advertisements in disguise are inadmissible. 3. In answering a query please quote the number. 4. If a private answer is desired, a stamped addressed envelope must be forwarded.

NOTICE.—We must really ask our correspondents to be more particular in enclosing name and address. Constantly we receive queries or letters with no name attached.

### Answers.

*Re Male Nurses.*—Please send name and address, and then we will gladly publish your letter.

*Southport Massage Institution.*—Will one or two old pupils of this institution kindly write to us in confidence.

(33) *St. Katherine's Hospital.*—This is at present in no way used as a home for nurses.

### In Memoriam.

ALICE FISHER, CHIEF NURSE OF THE PHILADELPHIA HOSPITAL.

(*Buried at Woodlands, June 5th*, 1888.)

IN a strange land a stranger! Low we laid her,
Coffin'd in English oak; her only pall
The flag 'neath which her sailor fathers conquer'd.
Like them, in stress of battle did she fall.

Only a stranger! Yet our city honours
With best and foremost sons this sad array;
And strong men weep, and the triumphant singing
Breaks into sobs of grief above her clay.

Only a stranger! With us four short winters.

"The English Nurse," men called her, as they smiled

In scorn that we should need her, soon forgetting

The friend they lov'd was not Columbia's child.

But now she is our own! For other strangers,
Our poor and sick, her very life she gave.
Oh! mother country, glorying in thy heroes,
She is our own for ever, by this grave.

"Woodlands" is a cemetery, the grounds of which overlook the hospital. At her express desire she was buried there, in a spot where, between the trees, the chimneys of the great city hospital can be plainly seen.

### Vacancies.

THE following vacancies are announced:

### Nurses.

Bethnal Green Guardians; £20.

Birkdale Home, Southport.

Blackheath and Richmond Institutions.

Borough Hospital, Birkenhead.

Bournemouth Institute; £25.

Bristol District Society; £42.

Brixton Hospital.

Cambridge Home.

Cheltenham Institution.

Children's Hospital, Pendlebury, Manchester; £20.

City and County Institution, Worcester.

District School Infirmary, Hanwell; £22.

Fleming Memorial Hospital, Newcastle-on-Tyne.

Hanover Institute, W.; £25.

Hooper's Agency; £50.

Hull Home.

Kidderminster Infirmary (night); £25.

Levick Institute, Paris.

Lock Hospital, Harrow Road, W.

Newport Institute; £20.

N.O.C. Hospital, Wisbech.

St. Saviour's Infirmary, Dulwich; £20.

Swansea and South Wales Institute; £25.

Ventnor (I.W.) Institution; £25.

Victoria Home, Bournemouth; £25.

Westhampnett Union; £22.

Workhouse Infirmary Association.

### Matrons.

Borough Hospital, Brighton; £60.

Cottage Hospital, Chalfont St. Peter's; £30.

National Hospital for Consumption, Ventnor; £70.

Wallasey Fever Hospital; £35.

### Probationers.

Borough Fever Hospital, Leeds.

Kendal Hospital; £10.

Workhouse Infirmary Association.

### Amusements and Relaxation.

### SECOND QUARTERLY WORD COMPETITION.

Commenced July 5th, 1890, ends Sept. 27th, 1890.

Three prizes of 15s., 10s., 5s., will be given for the largest number of words derived from the words set for dissection.

Proper names, abbreviations, foreign words, words of less than four letters, and repetitions are barred; plurals, and past and present participles of verbs, are allowed. Nuttall's *Standard* dictionary only to be used.

N.B.—Word dissections must be sent in WEEKLY not later than the first post on Thursday to the *Prize Editor*, 140, Strand, W.C., arranged alphabetically, with correct total affixed.

The word for dissection for this, the TENTH week of the quarter, being

"HARVEST."

| Names. | Aug. 28th. | Totals. |
|---|---|---|
| Dulcamara | ... | |
| Tinie | 23 | ... 524 |
| Henri | — | ... 395 |
| Agamemnon | 23 | ... 533 |
| Patience | 23 | ... 525 |
| Brisbane | — | ... 332 |
| Nurse Hilda | 23 | ... 505 |
| Lightowlers | 24 | ... 519 |
| Jenny Wren | 23 | ... 485 |
| Rhoda | — | ... 242 |
| Annabel | — | ... 17 |
| Spare Moments | — | ... 19 |
| Qu'appelle | 24 | ... 493 |
| Nurse | — | ... 29 |
| A. B. C. | — | ... 251 |
| Brickbat | — | ... 77 |

### Notice to Correspondents.

N.B.—Each paper must be signed by the author with his or her real name and address. A *nom de plume* may be added if the writer does not desire to be referred to by us by his real name. In the case of all prize-winners, however, the real name and address will be published.

Competitors can enter for *all* quarterly competitions, but no competitor may take more than *one first prize* during the year.

